# Association between group B beta-hemolytic *Streptococcus* screening during pregnancy and the prevalence of early-onset neonatal sepsis

**DOI:** 10.1590/1806-9282.20250077

**Published:** 2025-10-27

**Authors:** Thamirys Pereira Rodrigues, Marianna Camilo Rezende, Isadora Acerbi Manfrin, Edward Araujo, Alberto Borges Peixoto

**Affiliations:** 1 Universidade de Uberaba, Mario Palmério University Hospital, Gynecology and Obstetrics Service – Uberaba (MG), Brazil.; 2 Universidade Federal de São Paulo, Escola Paulista de Medicina, Department of Obstetrics – São Paulo (SP), Brazil.; 3 Universidade Municipal de São Caetano do Sul, Discipline of Woman Health – São Caetano do Sul (SP), Brazil.; 4 Universidade Federal do Triângulo Mineiro, Department of Obstetrics and Gynecology – Uberaba (MG), Brazil.

**Keywords:** Screening, Streptococcus, Neonatal sepsis, Preterm birth, Preterm premature rupture of the membranes, Chorioamnionitis

## Abstract

**OBJECTIVE:**

The aim of the study was to evaluate the incidence of early-onset neonatal sepsis and other perinatal adverse outcomes associated with not screening for group B beta-hemolytic *Streptococcus*.

**METHODS:**

A retrospective cohort study was conducted by searching electronic medical records from 2018 to 2022. Group B beta-hemolytic *Streptococcus* culture was performed after routine collection of vaginal and anal swabs from pregnant women at any time of pregnancy..

**RESULTS:**

A total of 968 pregnant women were included; 69.3% (675/968) were screened for group B beta-hemolytic Streptococcus, and 30.3% were not screened for group B beta-hemolytic *Streptococcus*. Of the pregnant women who were screened, 30.5% (206/675) had positive cultures and 69.5% (469/675) had negative cultures for group B beta-hemolytic *Streptococcus*. Pregnant women who underwent group B beta-hemolytic *Streptococcus* screening had a lower prevalence of preterm birth (p<0.0001), neonatal intensive care unit (NICU) admission (p=0.001), and neonatal death within 48 h (p=0.002). Group B beta-hemolytic *Streptococcus* screening was an independent predictor of preterm birth (p<0.0001). The best model for neonatal death in the first 48 h included group B beta-hemolytic *Streptococcus* screening (p=0.035) and NICU admission (p=0.016). Antibiotic use (p=0.040), preterm birth (p<0.0001), premature rupture of ovular membranes (p=0.047), premature delivery (p<0.0001), and chorioamnionitis (p=0.001) were associated with an increased risk of early-onset neonatal sepsis.

**CONCLUSIONS:**

Screening for group B beta-hemolytic *Streptococcus* was not significantly associated with early-onset neonatal sepsis, but it was an independent predictor of preterm birth.

## INTRODUCTION

Neonatal sepsis is a clinical entity of great importance due to its high morbidity and mortality. Despite advances in fetal medicine in recent decades, neonatal sepsis remains a challenge for obstetricians and pediatricians, and measures to prevent it are important to reduce adverse perinatal outcomes^1^. The major etiologic agent associated with neonatal sepsis is group B beta-hemolytic *Streptococcus* (GBS), also known as *Streptococcus agalactiae*. It is a gram-positive coccus that colonizes the ­genital and gastrointestinal tracts of 15–30% of pregnant women, and the vertical transmission rate is approximately 35–69%^2^.

Vaginal colonization by GBS was first described in 1935 by Lancefield and Hare^3^, but the first description of GBS-associated neonatal sepsis was in 1964 by Eickhoff et al.^4^. Since then, neonatal sepsis caused by GBS has become the leading cause of infectious morbidity and mortality worldwide^5^. In pregnant women, GBS infection is mostly asymptomatic; when associated with clinical signs and symptoms, it is responsible for asymptomatic bacteriuria and urinary tract infections. In neonates with GBS infection, the clinical presentation is associated with respiratory disease (54%), non-focal sepsis (27%), and meningitis (15%)^6^.

GBS vertical transmission occurs during passage through the birth canal or by aspiration of amniotic fluid infected with ascending GBS during prolonged premature rupture of the ovular membranes (PROM). Approximately 50 to 75% of newborns exposed to GBS become colonized, and 1 to 2% of infants of colonized mothers develop neonatal sepsis^7^. Risk factors for neonatal sepsis include GBS bacteriuria during pregnancy, gestational age <37 weeks, previous newborn with GBS infections, preterm birth, birth weight <2,500 g, prolonged labor, PROM, and maternal infection, including chorioamnionitis, bacteremia, sepsis, urinary tract infection, and maternal fever during labor^8^.

Given the adverse outcomes associated with neonatal sepsis caused by GBS infection, the objective of this study was to assess the true benefit of performing a GBS screening during prenatal care.

## METHODS

This study was a secondary analysis of a retrospective cohort, carried out at the Mario Palmério Hospital through a search of electronic medical records from 2018 to 2022. The project was approved by the Research Ethics Committee of the University of Uberaba – UNIUBE (CAAE: 52299421.7.0000.5145).

The inclusion criteria were the following: (1) pregnant women undergoing vaginal delivery and cesarean section at the service who have had a GBS screening at any time of pregnancy; (2) pregnant women undergoing vaginal delivery and cesarean section at the service who have not had a GBS screening; (3) absence of chromosomal disorders or congenital malformations diagnosed during prenatal care or after delivery.

The pregnant women who did not have a GBS screening during pregnancy were those whose prenatal care was provided by the Uberaba Municipal Health Department, where culture for GBS is not recommended.

GBS culture was performed after routine collection of vaginal and anal swabs from pregnant women attending the service between 35 and 37 weeks of gestation. A GBS culture was also obtained from pregnant women admitted to the obstetric unit with a diagnosis of preterm birth and/or PROM, regardless of gestational age at the time of diagnosis. To collect the swab, pregnant women were asked not to have bathed, douched, or used topical vaginal medications on the day of testing and not to have had sexual intercourse in the previous 24 h. To collect the vaginal sample, a swab was inserted approximately 2 cm into the introitus and rotated to reach the entire circumference of the vaginal wall. An anal sample was then collected by inserting another swab 0.5 cm from the anal sphincter and rotating it to reach the entire circumference of the region. Immediately after collection, each swab was individually placed in a tube containing a transport medium called Stuart (Biocon^®^, Belo Horizonte, Brazil) and stored at room temperature until sent to the laboratory within a maximum of 3 days.

In our service, prophylactic antibiotics are indicated for all pregnant women with a positive culture for GBS on admission for induction or labor conduction, with the exception of pregnant women who will undergo elective cesarean section with intact ovular membranes. Prophylactic antibiotics are also indicated for all pregnant women admitted to the hospital with a diagnosis of preterm birth and/or PROM, the results of the culture taken at the time of admission. In this case, in the presence of a negative GBS culture, antibiotic prophylaxis is discontinued. In the presence of a positive GBS culture, antibiotic prophylaxis is continued for 7 days.

The antibiotic of choice is penicillin G crystalline at a dose of 5 million IU intravenous (IV) as a starting dose, followed by 2.5 million IU IV every 4/4 h until the time of delivery. If penicillin G crystalline is not available, ampicillin 2 g (IV) is given as a starting dose, followed by 1 g (IV) every 6 h until delivery. In cases of allergy to penicillin G crystalline, clindamycin 900 mg IV is given 8/8 h until delivery. For pregnant women undergoing cesarean section, cefazolin 2 g (IV) may also be given as a loading dose, followed by 1 g (IV) 6/6 h until the moment of fetal extraction. Administration of two doses of any antibiotic within 4 h of delivery is considered adequate prophylaxis^9^.

To characterize the study population, the following variables were assessed: maternal age, ethnicity, number of pregnancies, number of deliveries, weight, height, body mass index (BMI), smoking, alcohol consumption, pre-existing chronic diseases, screening or not for GBS, gestational age at the GBS screening, GBS culture result, gestational age at hospitalization, preterm birth, presence of PROM, duration of PROM, antibiotic use, type of antibiotic, number of doses of antibiotic.

The data were transferred to an Excel 2010 spreadsheet (Redmond, WA, USA) and then analyzed using Statistical Package for the Social Sciences (SPSS) 20.0 (Chicago, IL, USA) and GraphPad Prism 7.0 (San Diego, CA, USA). Quantitative variables were first subjected to a normality test (Kolmogorov-Smirnov), and those with a parametric distribution were presented as means and standard deviations. Non-parametrically distributed variables were presented as medians and minimum and maximum values. Categorical variables were described using absolute and percentage frequencies and presented in tables. The chi-squared test was used to examine differences between categorical variables and their proportions. Student's t-test or Mann-Whitney U test was used to study the effect of the study group on continuous variables. Binary logistic regression was used to assess the best predictors of early-onset neonatal sepsis. The significance level for all tests was p=0.05.

## RESULTS

**Figure 1:**
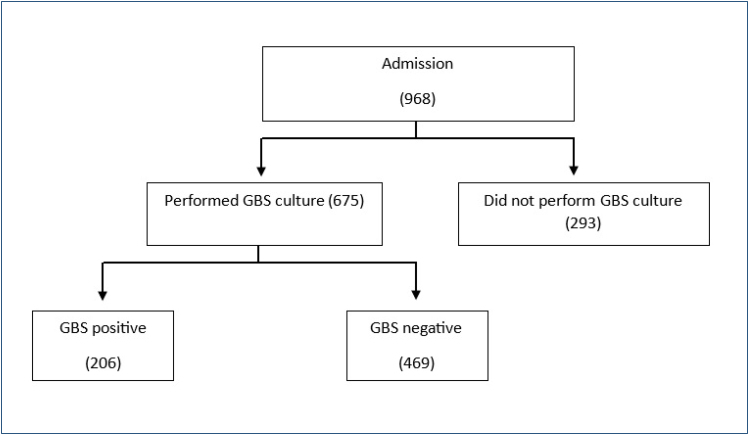
Flowchart of patients included in the study.

A total of 968 pregnant women were included; 69.3% (675/968) were screened for GBS (Group I), and 30.3% (293/968) were not screened for GBS (Group II). Of the pregnant women who were screened, 30.5% (206/675) had positive cultures and 69.5% (469/675) had negative cultures for GBS (Figure 1).

Group I had a higher prevalence of white ethnicity (46.9 vs. 37.2%, p=0.017), a lower prevalence of nulliparous pregnancy (9.0 vs. 14.0%, p=0.021), a higher prevalence of high-risk pregnancy (38.4 vs. 30.4%, p=0.017), higher prevalence of antibiotic use (35.5 vs. 10.9%, p<0.0001), lower prevalence of vaginal delivery (46.1 vs. 58.7%, p<0.0001). Group I had a lower median time of amniorrhexis (8.0 vs. 12.0 h, p=0.033), higher median gestational age at delivery (39.1 vs. 39.0 weeks, p=0.004), and higher median birth weight (3,200.0 vs. 3,115 g, p=0.003) (Table 1).

**Table 1:** Clinical characteristics of the population who underwent group B beta-hemolytic *Streptococcus* screening.

	Group I (675)	Group II (293)	p
Maternal age (years)	28.0 (14.0–49.0)	24.0 (14.0–43.0)	0.609†
Weight (kg)	77.8 (45.0–140.0)	69.8 (55.0–84.5)	0.359†
Height (m)	1.70 (1.50–1.80)	1.65 (1.60–1.70)	0.996†
BMI (kg/m^2^)	28.2 (19.5–44.8)	26.0 (21.0–31.0)	0.359†
**Ethnicity**			0.017§
White	46.9% (312/665)	37.2% (109/293)	
Black	8.4% (56/665)	12.3% (36/293)	
Mixed	43.8% (291/665)	50.2% (147/293)	
Asian	0.9% (6/665)	0.3% (1/293)	
Number of pregnancies	2.0 (1.0–8.0)	2.0 (1.0–7.0)	0.681†
Number of deliveries	9.0% (61/675)	14.0% (41/293)	0.021§
GA at GBS screening (weeks)	36.0 (21.7–37.0)		*
High-risk pregnancy	38.4% (259/675)	30.4% (89/293)	0.017§
Time of amniorrhexis (h)	8.0 (0.5–1,680)	12.0 (1.0–96.0)	0.033†
Use of antibiotics	35.5% (238/675)	10.9% (32/293)	<0.0001§
GA at delivery (weeks)	39.1 (22.0–41.6)	39.0 (21.3–41.3)	0.004†
**Type of delivery**			<0.0001§
Vaginal	46.1% (311/675)	58.7% (172/293)	
Cesarean section	53.6% (362/675)	40.3% (118/293)	
Forceps	0.3% (2/675)	1.0% (3/293)	
Birth weight (g)	3,200.0 (648.0–4,660)	3,115 (360–4,485)	0.003†
Apgar score at the 1st minute	9.0 (0.0–10.0)	8.0 (1.0–9.0)	0.275†
Apgar score at the 5th minute	9.0 (0.0–10.0)	9.0 (0.0–10.0)	0.200†

Group I: GBS screening and Group II: no GBS screening. BMI: body mass index; GA: gestational age; PROM: premature rupture of ovular membranes; GBS: group B beta-hemolytic *Streptococcus*. Mann-Whitney

† median (minimum–maximum); chi-squared

§ percentage (n/N);

* statistical test could not be used due to the absence of at least three cases in one of the groups. p<0.05.

Pregnant women who underwent GBS screening had a lower prevalence of preterm birth [12.4 vs. 24.6%, OR 0.44 (0.30–0.62), p<0.0001], neonatal intensive care unit (NICU) admission [7.0 vs. 13.3%, OR 0.49 (0.31–0.76), p=0.001], and neonatal death within 48 h [0.6 vs. 3.1%, OR 0.19 (0.06–0.61), p=0.002] (Table 2).

**Table 2:** Association between screening and non-screening for group B beta-hemolytic *Streptococcus* and adverse perinatal outcomes.

	Group I (675)	Group II (293)	OR (95% CI)	p
Preterm birth	12.4% (84/675)	24.6% (72/293)	0.44 (0.30–0.62)	<0.0001
PROM	20.6% (139/675)	25.6% (75/293)	0.75 (0.54–1.04)	0.085
Neonatal sepsis	2.5% (17/675)	3.1% (9/293)	0.81 (0.35–1.85)	0.625
Apgar score at the 1st minute <7	7.5% (50/671)	10.3% (30/292)	0.70 (0.43–1.13)	0.145
NICU admission	7.0% (47/675)	13.3% (39/293)	0.49 (0.31–0.76)	0.001
Neonatal death within the first 48 h	0.6% (4/675)	3.1% (9/292)	0.19 (0.06–0.61)	0.002
Chorioamnionitis	0.6% (4/675)	1.0% (3/293)	0.57 (0.13–2.59)	0.467
Maternal ICU admission	1.2% (8/675)	0.3% (1/293)	3.5 (0.43–28.12)	0.209

Group I: GBS screening and Group II: no GBS screening. CI: confidence interval; NICU: neonatal intensive care unit; OR: odds ratio; PROM: premature rupture of ovular membranes; ICU: intensive care unit. Percentage (n/N); Binary logistic regression. p<0.05.

A forward stepwise binary logistic regression model was constructed using GBS screening, PROM, antibiotic use, and correct antibiotic use to determine the best predictors of preterm birth. A positive culture for GBS was an independent predictor of preterm birth [χ^2^ 21.7 (1), R^2^ Nagelkerke: 0.12, OR 0.11, 95%CI 0.05–0.26, p<0.0001]. PROM (p=0.426), use of antibiotics (p=0.453), and correct use of antibiotics (p=0.054) were not significant predictors of preterm birth.

A forward stepwise binary logistic regression model was constructed using GBS screening, PROM, antibiotic use, correct antibiotic use, preterm birth, and NICU admission to determine the best predictors of neonatal death within the first 48 h. The best model was the one including positive culture for GBS [χ^2^ 12.3 (2), R^2^ Nagelkerke: 0.35, OR 0.06, 95%CI 0.005–0.82, p=0.035] and NICU admission [χ^2^ 12.3 (2), R^2^ Nagelkerke: 0.35, OR 22.83, 95%CI 1.81–288.12, p=0.016]. PROM (p=0.374), antibiotic use (p=0.956), correct antibiotic use (p=0.516), and preterm birth (p=0.661) were not significant predictors of neonatal death in the first 48 h.

It was observed that antibiotic use (OR 2.27, 95%CI 1.03–4.97, p=0.040), preterm birth (OR 7.74, 95%CI 3.48–17.21, p<0.0001), PROM (OR 2.26, 95%CI 1.01–5.05, p=0.047), premature delivery (OR 16.04, 95%CI 6.34–40.59, p<0.0001), and chorioamnionitis (OR 15.61, 95%CI 2.88–84.56, p=0.001) were associated with an increased risk of early-onset neonatal sepsis when considering all pregnant women who underwent GBS screening or not. Positive culture for GBS (p=0.253), correct use of antibiotics (p=0.207), and NICU admission (p=0.988) were not associated with a significant increase in the risk of early-onset neonatal sepsis (Table 3).

**Table 3:** Risk of early-onset neonatal sepsis considering all pregnant women who underwent group B beta-hemolytic *Streptococcus* screening or not.

Variable	OR (95%CI)	p
Positive culture for GBS	0.48 (0.13–1.69)	0.253
Use of antibiotics	2.27 (1.03–4.97)	0.040
Correct use of antibiotics	3.77 (0.47–29.83)	0.207
Preterm birth	7.74 (3.48–17.21)	<0.0001
PROM	2.26 (1.01–5.05)	0.047
Premature delivery	16.04 (6.34–40.59)	<0.0001
Chorioamnionitis	15.61 (2.88–84.56)	0.001
NICU admission	70.0 (0.00–infinite)	0.988

CI: confidence interval; NICU: neonatal intensive care unit; OR: odds ratio; PROM: premature rupture of ovular membranes; GBS: group B beta-hemolytic *Streptococcus*. Binary logistic regression. p<0.05.

## DISCUSSION

Neonatal sepsis is a clinical syndrome characterized by systemic signs of infection with the presence of bacteremia in the first month of life^10^. The early-onset form occurs in the first 24 h or up to the 7th day of life and accounts for 85% of neonatal infections. Neonates with early-onset disease usually present with respiratory distress, apnea, or other signs of sepsis. The most common clinical diseases of early-onset disease are ­sepsis and pneumonia. Less commonly, they can lead to meningitis, and approximately 15–30% of meningitis survivors have neurologic sequela^11^.

The medical literature indicates that maternal colonization with GBS is generally asymptomatic, reinforcing the need for universal screening between 35 and 37 weeks of gestation^8^. This procedure allows the administration of intrapartum antibiotic prophylaxis, which has been shown to be an effective strategy to reduce vertical transmission and consequently neonatal complications^1^. In the present study, the absence of GBS screening in a significant proportion of the pregnant women analyzed was associated with an increase in adverse perinatal outcomes.

Comparing the results of this study with the existing ­literature, it was observed that intrapartum prophylaxis based on appropriate screening is an essential tool in the prevention of neonatal sepsis. Studies such as Verani et al.^8^ and Shane et al.^1^ have shown that the introduction of systematic screening ­policies for GBS in countries such as the United States has been responsible for a significant reduction in early-onset neonatal sepsis rates. Similarly, the present study confirms the importance of this screening by showing that pregnant women who were not screened for GBS had higher rates of NICU admission and neonatal death in the first 48 h compared to those who were screened.

Prolonged PROM is widely recognized as a predisposing factor for the entry of bacteria into the intrauterine environment, increasing the risk of neonatal sepsis^12,13^. This study highlights that inappropriate antibiotic use associated with preterm birth, PROM, premature delivery, and chorioamnionitis was ­associated with a higher rate of early-onset neonatal sepsis, confirming the need for strict obstetric surveillance in these situations.

Another issue discussed in this study is the effectiveness of intrapartum antibiotic prophylaxis. Although prophylaxis is recommended for all pregnant women with a positive culture for GBS^9^, the correct use of antibiotics is not always implemented according to established protocols, which may compromise the effectiveness of the intervention. In this context, the study shows that even among pregnant women with a GBS-positive culture, not all received adequate antibiotic prophylaxis, highlighting the importance of continuous education of healthcare teams and the implementation of robust institutional protocols to ensure the correct administration of antibiotics, preferably within 4 h of delivery.

Antibiotic use, preterm birth, and PROM are significant predictors of neonatal complications already known in the literature^14,15^, as shown by the logistic regression models presented in the present study. However, this study found that screening for GBS did not have a statistically significant association with the prevalence of early-onset neonatal sepsis. This suggests that although GBS screening is widely recommended to reduce infectious complications in newborns, its implementation alone may not be sufficient to prevent neonatal sepsis, especially when other risk factors are present. However, a positive culture was identified as an independent predictor of preterm birth, a finding of great importance because it reinforces the association between subclinical perinatal infection and prematurity, which is itself an important determinant of neonatal morbidity and mortality. Furthermore, when the variable NICU admission was included in the model as a confounder, the risk initially associated with GBS screening in relation to neonatal death within the first 48 h was reduced. This suggests that the NICU admission may act as a moderating factor, influencing more serious outcomes and reducing the isolated importance of GBS screening in preventing early neonatal mortality.

## CONCLUSION

Screening for GBS in pregnant women has been associated with improved perinatal outcomes, including lower rates of preterm birth, NICU admission, and neonatal death within the first 48 h of life. A positive GBS culture has been identified as an independent predictor of preterm birth, while the combination of a positive GBS culture and NICU admission has been shown to be the best predictor of early neonatal death. Factors such as antibiotic use, preterm birth, PROM, and chorioamnionitis have been significantly associated with an increased risk of early-onset neonatal sepsis. However, a positive GBS culture, appropriate antibiotic use, and NICU admission were not significantly associated with this outcome. These findings underscore the importance of universal GBS screening during prenatal care for targeting preventive interventions and improving neonatal outcomes. Nevertheless, the presence of additional obstetric and infectious factors necessitates continued attention and individualized protocols for the prevention of neonatal sepsis.

## Data Availability

The datasets generated and/or analyzed during the current study are available from the corresponding author upon reasonable request.
